# Subjective Response Measurement to Prosthesis or Device Use: Validation of the Prosthetic–Bionic Paradigm Questionnaire (PBP-Q)

**DOI:** 10.3390/ijerph19084656

**Published:** 2022-04-12

**Authors:** Augusto Iossa Fasano, Paolo Mandolillo, Yura Loscalzo, Marco Giannini, Gabriele Grippo, Isabella Imbimbo, Rosapia Lauro Grotto

**Affiliations:** 1 Cultural Scientific Association “Metandro”, 51100 Pistoia, Italy; info@augustoiossafasano.it (A.I.F.); paolomandolillo@libero.it (P.M.); 2 School of Psychoanalytic and Group Analytic Psychotherapy SPPG, 89100 Reggio Calabria, Italy; 3 Department of Health Sciences, School of Psychology, University of Florence, 50135 Florence, Italy; marco.giannini@unifi.it (M.G.); rosapia.laurogrotto@unifi.it (R.L.G.); 4 Cardiology Department, Ospedale di Prato, 59100 Prato, Italy; grippogabriele1978@yahoo.it; 5 Clinical Psychology Unit, Fondazione Policlinico Universitario A. Gemelli IRCCS, 00100 Rome, Italy; isabellaimbimbo@gmail.com; 6 Department of Aging, Neurological, Orthopedic, and Head-Neck Sciences, Fondazione Policlinico Universitario A. Gemelli IRCCS, 00100 Rome, Italy; 7 Multidisciplinary Laboratory for the Analysis of Relationships in Health Care (M.A.R.H.C. Lab.), University of Pistoia Uniser, 51100 Pistoia, Italy

**Keywords:** prosthesis, device, prosthetic–bionic paradigm, identity, technological medicine

## Abstract

Many subjects with somatic pathologies or traumas in their recent anamnesis tend to experience symptoms and changes to their daily life parameters after technically successful treatment. Hence, this study aims to validate an investigation tool inspired by the prosthetic–bionic paradigm—namely, the PBP-Q—which allows for the evaluation of variation in questions relating to identity, psychosociality, and psychopathology in relation to the use of a prosthesis or device. We gathered 118 participants (68 females and 50 males) aged between 27 and 94 years (Mean = 58.42 ± 15.17). We performed both exploratory (EFA) and confirmatory (CFA) factor analyses on this sample. Moreover, we calculated the internal consistency for the PBP-Q scales and the total score for the questionnaire’s final 26-item and 5-factor versions. The five scales are psychological well-being; interpersonal relationships; professional relationships; autonomy and safety; addictions, compulsions, and obsessions. The internal consistency is good for both the total score and the subscales. In conclusion, overall, the PBP-Q has satisfactory psychometric properties, especially considering the measure’s complexity. It provides a quick and effective way to evaluate the changes that might arise after the use of a prosthesis or device and, subsequently, has implications for clinical practice.

## 1. Introduction

Since the postwar period, contemporary medicine has increasingly turned to biomedical and bioengineering technology. Every year, many people worldwide receive medical and surgical interventions involving the grafting of devices or prostheses to support bodily functions that are deficient or that have been altered or permanently lost. Although these medical interventions can result in clinical recovery on the somatic level, they do not always have good outcomes in what concerns the psychic well-being and the overall socio-environmental health of patients. Many subjects with a recent anamnesis of somatic pathologies (heart disease, cancer, neurodegenerative pathologies) or trauma (amputations, prosthetic implants, plastic surgery) experience changes in their daily life once their technically successful treatment is finished. They report symptoms such as loss of sense of identity and existential continuity, anxiety, panic, insomnia, obsessive ideation and impulsiveness, mood alterations, and acute or chronic post-traumatic stress disorders. The literature reports cases of reduced quality of life for subjects with pacemakers [1], poor assimilation of and low compliance to upper limb prosthetic devices, regardless of age and gender [2,3], and powerful emotional responses ranging from euphoria to despair associated with exoskeleton-related paraplegia [4]. For those patients adopting deep brain stimulators (DBSs), the following responses have been observed: increased depression, apathy, or impulsivity in patients with Parkinson’s disease [5], as well as an increased risk of suicide or suicidal ideation [6]. Despite the downsides associated with prostheses, however, the literature about the negative psychological effects of these devices is scant and primarily qualitative, probably due to the heterogeneity of the samples relating to premorbid psychological issues [7,8]. Additionally, even if some studies on psychological issues associated with prostheses, including limb ones, have adopted a quantitative perspective [9], a unified theoretical model, supported by empirical evidence, has yet to be designed. On the other hand, the literature on the positive impact of psychological interventions addressed to people with devices is wider. These include many different clinical contexts, such as the implantation of ventricular support devices (VAD) [10,11], DBS for Parkinson′s and severe neuropsychiatric pathologies [12], limb prostheses for amputees [13], and people with congenital deficiencies [14,15], breast implants for women with cancer [16], ocular prostheses to counteract eye defects or ptosis of the eyelid [17], dental prostheses [18], and even exoskeletons for patients with spinal cord injuries [19].

Overall, conceptual analysis of the impact of prostheses or devices on individual psychological structures remains insufficient. It is of paramount importance to study this phenomenon from a perspective that can deepen our understanding of how psychological interventions can help ensure adaptation to a given device and achieve long-term improvements in quality of life during the post-intervention phase. In this regard, it is useful to move beyond the Cartesian dualism of res extensa and res cogitans, choosing instead an approach informed by the concept of embodiment. Embodiment is a unitary conception according to which the structure of the mind is always in relation to the body. In brief, the functions of the mind are inseparable from those of the body. Embodiment is a construct increasingly used in neuroscience studies that try to identify its neural imprint [20,21], by psychologists who try to refine its definition [22,23], and by biomedical engineers working in the field of rehabilitation [24].

Although it is clear that prostheses and devices modify the representations of the human body and peri-personal space [25,26], more profound reflection is necessary when it comes to the concept of identity. In the present study, we understand identity as a composite and changing psychological element, one that is closely associated with the device or prosthesis (terms used as synonyms in this paper in a reductionist perspective, foreseeing subsequent developments of research on the differentiation of the types of prostheses/devices and of subjective reactions in the various specialist fields). Identity is composite, as it is made up of three components (i.e., mind, body, and prosthesis), and it is mutant because it continually changes throughout an individual’s lifetime. In recent years, Iossa Fasano [27] has developed an innovative proposal derived from a Freudian perspective, one that draws attention to the fact that in the psychoanalytic model itself the investment (cathexis) of the body’s functioning constitutes a fundamental process that continuously structures and restructures the mental dimension [28,29], providing the integrative basis of Ego processes.

The prosthetic–bionic paradigm (PBP) [27,30,31] claims that, in the contemporary world, it is necessary to postulate an evolution of the human toward a cyborg identity. Prosthetic integration points to a hybrid structure of identity, which is conceived as a “spontaneous” dimension that occurs through the body along the external–internal continuum [27]. This model defines human identity as prosthetic since psychic functioning involves the body–device interaction. Such structural interaction occurs between the subject and an auxiliary object that acts as a medium with the external world. This, therefore, demonstrates the structurally vulnerable nature of the body, and this deficit influences the constitution and development of the psychic apparatus. This situation is invisible (or unconscious) for healthy subjects, but the occurrence of disease, structural loss of an organ or limb, or loss of a function highlight the difficulties inherent in the maintenance or recovery of identity. Devices and prostheses (outside or inside the body) are applied to subjects who understand themselves as in the process of trying to maintain their identities or trying to recover “who I am”. Physical treatment alone may not be sufficient for a successful achievement of this psychosocial recovery.

Over the last 15 years, the Metandro Association conducted in various Italian hospitals and universities (Milan, Prato, Rome, and Pistoia) numerous exploratory investigations about the subjective response of patients to new high-tech treatment methods, such as the implantation of internal devices or use of external prostheses (wearable technology). Clinical observation has shown frequent anxiety spectrum symptoms (from acute to chronic post-traumatic stress) in a relevant subgroup of users who have received such medical-surgical treatments. Starting from these practical experiences, we set ourselves the goal of understanding if, and to what extent, devices affect identity, ordinary relationships, and psychopathological dimensions such as anxiety, depression, obsessive thoughts, and compulsive behaviors. These are psychological factors that, if present, might compromise the subject’s adoption of prostheses and the overall compliance, with negative consequences for the person’s quality of life, but also for the person’s life itself, given that most of the devices are lifesavers. More specifically, we were motivated by one central question: Is it possible to determine the extent to which high-tech, innovative medical–surgical treatments affect a person’s life beyond the technical success of the intervention? To answer this question, we developed a pool of 65 items for a questionnaire founded on the PBP model. Next, to create a short version with good psychometric properties for both clinical practice and research purposes, we administered it to over 100 prostheses or device users in the last five years, aiming to conduct factorial and reliability analyses. Hence, this research aims to present in the literature the Prosthetic–Bionic Paradigm Questionnaire (PBP-Q) as a screening and assessment tool capable of analyzing the influence that different types of prostheses or devices can have on personal, identity, and relational levels, as well as their possible psychopathological consequences.

## 2. Materials and Methods

### 2.1. Participants

The sample is made up of 118 participants (68 females and 50 males) with a mean age of 58.42 (SD = 15.17; extensive range: 27–94 years). The sampling method was not a fixed or predefined selection process (i.e., non-probability sampling), and the data were collected by recruiting patients throughout the Italian territory. The level of education ranges from primary school to post-graduate specialization. The participants are also involved in different types of occupations. The sample is also heterogeneous regarding prostheses, including mostly cardiological, dental, orthopedic, and neurosurgical devices.

### 2.2. Materials

The participants filled out the 65-item PBP-Q, a tool designed to assess the impact of prostheses on quality of life. The PBP-Q arose in the context of psychoanalysis treatment addressed to patients with prostheses and devices due to limb amputations, transplants, heart disease, and tumors. Hence, we aimed to design a questionnaire for the investigation of various parameters of adaptation (not only to the disease or to the technological cure). The PBP-Q (see Appendix A) consists of three sections: (i) personal information (gender, age, educational qualification, profession); (ii) anamnestic collection (risk factors, past pathological anamnesis, next pathological anamnesis, pharmacological anamnesis); (iii) bionic-prosthetic (BP) interview asking about the devices currently used or used over the years. The areas of application are aesthetic medicine/surgery, orthopedics, dentistry and implantology, organ transplantation, cardiology, pumps and infusoria, urogenital system, ophthalmology–acoustics, neurosurgery, and medicine and general surgery.

The pool of 65 items was designed based on the observation and treatment of patients with prostheses or internal devices, paying particular attention to four dimensions capable of favoring or compromising compliance with care pathways: (i) in total, 33 items investigate the changes observed since the person adopted the prosthesis in the personal, interpersonal and professional sphere; (ii) in addition, 11 items evaluate to what extent the prosthesis influenced or modified psychological and existential experiences; (iii) another 16 items address the consequences deriving from the use of the prosthesis; (iv) lastly, 5 items investigate the acceptance and recognition of an improvement or deterioration in the quality of life. In addition to this quantitative part, the pilot version of the PBP-Q also includes a qualitative section, which consists of five open questions asking the subjects about their personal experiences relating to the device, as well as three questions for the questionnaire administrator. Since the present paper focused on the PBP-Q psychometric properties, the qualitative part was not used for statistical analysis in the current study.

### 2.3. Procedure

The data were collected by recruiting patients from various clinical centers distributed throughout the Italian territory, including the Hemodynamics Service of the Prato Hospital (with the approval of the Regional Ethics Committee for Clinical Trials of the Tuscany Region) and the Department of Neurology of the “Gemelli” Polyclinic in Rome, in the period between 2016 and 2020. The test was administered by the psychologists of the Metandro Association, who were suitably trained in the presentation of informed consent, the purposes of the research, and the questionnaire itself. The patients’ doctors were also informed.

### 2.4. Data Analysis

First, we conducted an exploratory factor analysis (EFA) with the principal axis method and Promax rotation on the 65-item version of the PBP-Q (i.e., Section 3), aiming to reduce the number of items on the scale. Next, we analyzed the descriptive statistics and the internal consistency of the PBP-Q scales, and we performed a *t*-test and ANOVA to evaluate if there are differences in the PBP-Q scales concerning gender and the three types of prosthetic rehabilitation (i.e., orthopedic prosthesis, dental prosthesis, and cardiological device). Finally, we cross-validated the EFA factor structure through confirmatory factor analysis (CFA; maximum likelihood estimation). To evaluate the fit of the model, we used the following indices: χ2/df ratio, which indicates a good fit if its value is less than 3 [32]; however, it should be noted that this value is influenced by the sample size [33]; goodness of fit index (GFI), comparative fit index (CFI), and Tucker-Lewis index (TLI), which have the following cut-off values: <0.90 lack of fit, 0.90–0.95 good fit, >0.95 excellent fit [34,35]; the root mean square error of approximation (RMSEA), for which a value below 0.05 is indicative of an excellent fit, while a value between 0.05 and 0.08 indicates an acceptable fit [35,36].

## 3. Results

First, we performed EFAs on the 65-item pilot version of the PBP-Q to reduce the number of items and have at least four items for each factor. Hence, we reached a 26-item and 5-factor version. For this final solution, the first factor explains 27.94% of the variance, while the cumulative variance values explained, respectively, by the second, third, fourth, and fifth factors are 38.44%, 47.17%, 54.41%, and 60.58%. Referring to the content of the items belonging to the same factor, we labeled them: (i) psychological well-being; (ii) interpersonal relationships; (iii) professional relationships; (iv) autonomy and safety; (v) addictions, compulsions, and obsessions. Table 1 shows the EFA results and the internal reliability for the PBP-Q scales, as calculated on the total sample. The α value for the total score is 0.85.

Using *t*-test analysis, no significant gender differences were found on the PBP-Q scales (psychological well-being: *t*(1,108) = 0.004, *p* = 0.996; interpersonal relationships: *t*(1,108) = 0.138, *p* = 0.086; professional relationships: *t*(1,108) = −0.47, *p* = 0.642; autonomy and safety, *t*(1,108) = −1.06, *p* = 0.292; addictions, compulsions, and obsessions: *t*(1,108) = −1.04, *p* = 0.300). Moreover, the ANOVA test did not show statistically significant difference in the PBQ-Q scales regarding the three types of prostheses (psychological well-being: *F*(2,88) = 1.70, *p* = 0.188; interpersonal relationships: *F*(2,88) = 0.39, *p* = 0.680; professional relationships: *F*(2,88) = 1.48, *p* = 0.233; autonomy and safety: *F*(2,88) = 1.23, *p* = 0.297; addictions, compulsions, and obsessions: *F*(2,88) = 0.24, *p* = 0.786).

Table 2 shows the descriptive statistics of the PBP-Q five scales obtained by all the participants in the study (*n* = 118).

**Figure 1:**
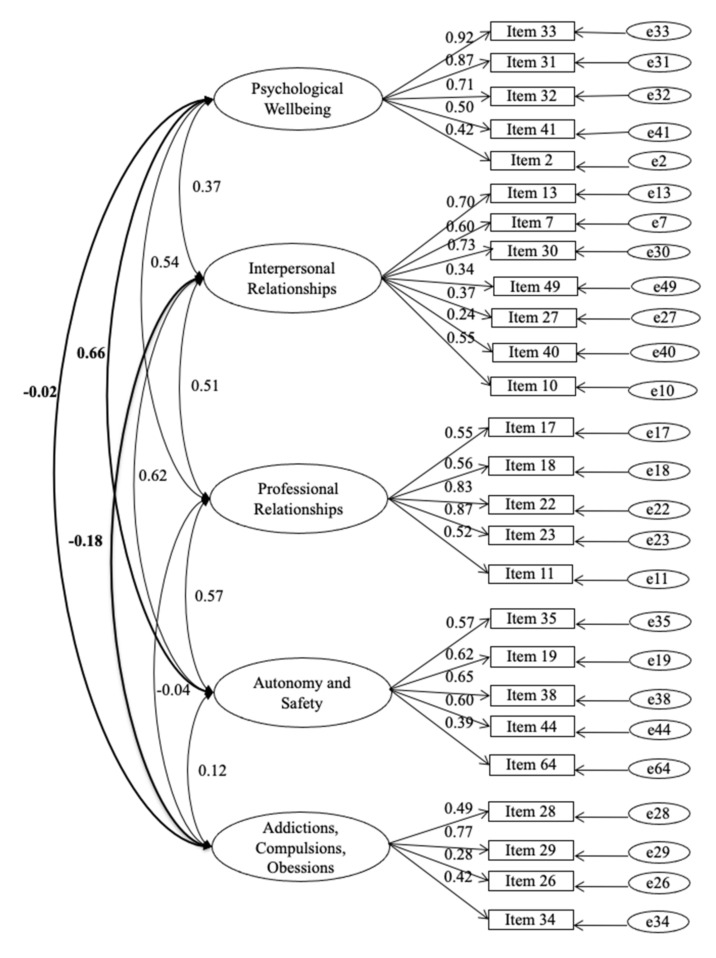
Five-Factor Model, Prosthetic Bionic Paradigm-Questionaire (PBP-Q), *n* = 118.

Finally, we performed CFA (*n* = 118), aiming to cross-validate the factor structure we found through the EFA. In line with the complexity of the construct, the fit indexes are not good for the 5-factor model and 26-item solution: χ2 = 647.073, df = 289, χ2/df = 2.24, *p* < 0.001; GFI = 0.72; CFI = 0.68; TLI = 0.64; RMSEA = 0.103 (C.I. 90% = 0.092–0.114). However, all of the standardized factor loadings are statistically significant and range between 0.24 and 0.92. Figure 1 shows the graphical representation of this model.

## 4. Discussion

In recent decades, impressive results have been achieved in developing new prosthetic technologies and devices that recover functionality for people who have lost a body part or function. Despite this technological progress, understanding the psychological difficulties implied by these interventions and the personalized treatment required is still a challenge. The chance to explore the complexity of the identity changes related to the presence of devices within the body offers a remarkable opportunity for comprehension and healing, as it allows us to focus on peculiarities and differences, hence avoiding discrimination within a heterogeneous category that deserves a scientific approach (the Prosthetic–Bionic Paradigm) and, consequently, scientific investigation. Our clinical and research experience suggests that changes in subjectivity and identity among people with prosthetic devices must be carefully evaluated, so as to identify the domains in which there is the greatest risk of destabilization, in this way supporting compliance and helping to ensure the best possible post-surgery outcomes. It is for these reasons that we developed and validated the Prosthetic–Bionic Paradigm Questionnaire (PBP-Q), a new test aiming to describe and evaluate the psychological impact of the implantation within or on the body of prostheses/devices.

From the 65-item pilot version of the PBP-Q, using exploratory factor analysis, we reached a 5-factor structure including 26 items. Based on the content analysis of the items in the five subdimensions, we labeled the scales as follows: (i) psychological well-being; (ii) interpersonal relationships; (iii) professional relationships; (iv) autonomy and safety; (v) addictions, compulsions, and obsessions. Next, confirmatory factor analysis on the 5-factor and 26-item version of the PBP-Q showed that the global fit of the model is not entirely satisfactory, due to the complexity of the construct. However, all of the items have good and statistically significant loadings on the related factor, ranging between 0.24 and 0.92. Finally, the internal reliability of the subscales and the total score is high (for the total score, α = 0.85).

Therefore, the final version of the PBP-Q is composed of 26 items, which allows for a quick assessment of 5 dimensions: psychological well-being; interpersonal relationships; professional relationships; autonomy and safety; addictions, compulsions, and obsessions. High scores in the first four dimensions point to relevant changes in subjective identity related to the device implant, while high scores in the last scale point to the need to investigate possible psychopathological reactions that could merit psychological treatment. Evaluating the specific subdimensions of the PBP-Q can be helpful to discriminate among patients as well as to calibrate, within a selective counseling approach, the best psychological interventions, taking into account the patient’s interpersonal skills and potential for adaptation. We would also like to stress the relevance of exploring the indices of pathological risks or the intrusion of prostheses in the daily life experience of the patients: adaptation is neither a mandatory nor a rapid phenomenon; it is a gradual and articulated process that implies different dimensions of a patient’s biopsychosocial functioning that can be assessed with the PBP-Q.

The theoretical model of the prosthetic–bionic paradigm has been progressively corrected and revised according to the evidence collected during many years of clinical experience (counseling, rehabilitation, psychotherapy, psychoanalysis, and supervision of doctors, psychologists, and other practitioners). In light of the proposed framework, the current study provides evidence concerning identity adjustment in the aftermath of device utilization and is a useful instrument that can be employed in future studies to analyze further the PBP model and the effect of prostheses and devices on individuals. Moreover, as part of our data collection process, we collected a large set of qualitative data through the open questions section of the PBP-Q. Patients were keen to elaborate on the issues raised by the text-written items, and we plan to investigate this qualitative evidence in further studies.

In summary, in the face of the current developments in contemporary medicine, we suggest that future studies will be necessary to investigate the specific problems related to each of the prosthetic domains and therapeutic methodologies, giving consideration to not only the body of the patients but their overall sense of identity.

## 5. Conclusions

In the present study, the psychometric properties of the PBP-Q were evaluated. The PBP-Q is a self-report multidimensional test devoted to assessing, within a psychodynamic perspective, subjective response and identity-change-related prosthesis or device. A 26-item solution emerged from an exploratory factor analysis, including the following 5 scales: psychological well-being; interpersonal relationships; professional relationships; autonomy and safety; addictions, compulsions, and obsessions. The solution was further validated with confirmatory factor analysis. The availability of a reliable and reasonably quick measure of the psychological impact of the use of prostheses/devices can support further studies in the specific subdomains of this fast-developing medical context, providing the basis for tailored interventions aiming to support the complex psychological adaptation process of the patients.

## Figures and Tables

**Table 1:** Exploratory factor analysis (EFA) of the Prosthetic–Bionic Paradigm Questionnaire (PBP-Q; *n* = 118).

PBP-Q Item *Change/Impact on*…	PW	IR	PR	AS	ACO
33. Frequency of sexual activity	0.98				
31. Quality of sexual activity	0.79				
32. Sexual desire	0.78				
41. Satisfaction with weight	0.65				
2. Quality of sleep	0.35			0.32	
13. Quality of family relationships		0.82			
7. Quantity of social relations		0.68			
30. Quality of couple relationship/potential partner		0.62			
49. Perception of being more observed		0.54			
27. Care of personal appearance		0.41		0.33	
40. Appetite		0.39			
10. Esteem of others towards you		0.38		0.32	
17. The way your superiors behave towards you			0.94		
18. The way your colleagues treat you			0.83		
22. Quantity of professional relations			0.60		
23. Quality of professional relations			0.56		
11. Work opportunities and career prospects			0.36	0.31	
35. Autonomy				0.75	
19. Time dedicated to work				0.56	
38. Sense of security				0.56	
44. Interference with domestic abilities				0.54	
64. Improved quality of life thanks to the prosthesis				0.39	
28. Recurrent thoughts					0.74
29. Acting out repetitive or unnecessary actions					0.56
26. Consumption of substances					0.48
34. Quantity of medications					0.44
α (*n* = 118)	0.82	0.70	0.84	0.71	0.55

Note. Principal axis factoring, Promax rotation; factor loadings below 0.30 are not presented; PW = psychological well-being; IR = interpersonal relationships; PR = professional relationships; AS = autonomy and safety; ACO = addictions, compulsions, and obsessions. The number of the items reflects the order of the 65-item pilot version.

**Table 2:** Descriptive statistic of the Prosthetic–Bionic Paradigm Questionnaire (PBP-Q; *n* = 118) scales.

PBP-Q Scale	Num. Items	Min-Max	M(SD)
Psychological well-being	5	5–25	14.57 (3.55)
Interpersonal relationships	7	15–33	21.50 (3.76)
Professional relationships	5	11–25	16.14 (2.64)
Autonomy and safety	5	7–25	16.67 (3.48)
Addictions, compulsions, and obsessions	4	7–19	12.38 (2.26)

## Data Availability

The dataset is available, upon reasonable request, and for research purposes only, by writing to the corresponding author.
